# Switching between Ultrafast Pathways Enables a Green-Red Emission Ratiometric Fluorescent-Protein-Based Ca^2+^ Biosensor

**DOI:** 10.3390/ijms22010445

**Published:** 2021-01-05

**Authors:** Longteng Tang, Shuce Zhang, Yufeng Zhao, Nikita D. Rozanov, Liangdong Zhu, Jiahui Wu, Robert E. Campbell, Chong Fang

**Affiliations:** 1 Department of Chemistry, Oregon State University, 153 Gilbert Hall, Corvallis, OR 97331-4003, USA; tanglo@oregonstate.edu (L.T.); rozanovn@oregonstate.edu (N.D.R.); zhul@oregonstate.edu (L.Z.); 2 Department of Chemistry, University of Alberta, Edmonton, AB T6G 2G2, Canada; shuce@ualberta.ca (S.Z.); yufeng@ualberta.ca (Y.Z.); jiahui5@ualberta.ca (J.W.); robert.e.campbell@ualberta.ca or; 3 Department of Chemistry, Graduate School of Science, The University of Tokyo, 7-3-1 Hongo, Bunkyo-ku, Tokyo 113-0033, Japan

**Keywords:** red fluorescent protein based Ca^2+^-biosensor, photochemistry, ultrafast dynamics, structure-activity relationships, cell imaging

## Abstract

Ratiometric indicators with long emission wavelengths are highly preferred in modern bioimaging and life sciences. Herein, we elucidated the working mechanism of a standalone red fluorescent protein (FP)-based Ca^2+^ biosensor, REX-GECO1, using a series of spectroscopic and computational methods. Upon 480 nm photoexcitation, the Ca^2+^-free biosensor chromophore becomes trapped in an excited dark state. Binding with Ca^2+^ switches the route to ultrafast excited-state proton transfer through a short hydrogen bond to an adjacent Glu80 residue, which is key for the biosensor’s functionality. Inspired by the 2D-fluorescence map, REX-GECO1 for Ca^2+^ imaging in the ionomycin-treated human HeLa cells was achieved for the first time with a red/green emission ratio change (ΔR/R_0_) of ~300%, outperforming many FRET- and single FP-based indicators. These spectroscopy-driven discoveries enable targeted design for the next-generation biosensors with larger dynamic range and longer emission wavelengths.

## 1. Introduction

Tracking intracellular Ca^2+^ is of paramount importance to cell biology since Ca^2+^ is a ubiquitous second messenger that regulates numerous cellular processes [1]. Ever since the discovery of green fluorescent protein (GFP) [2], FP-based genetically encoded Ca^2+^ indicators, which include the Förster resonance energy transfer (FRET) and GCaMP types, have been actively developed to achieve a large dynamic range, Stokes shift, and long emission wavelengths [3,4,5,6,7]. In applications, ratiometric (excitation or emission) imaging is preferred due to the elimination of undesirable effects caused by complex experimental conditions such as varied expression levels, uneven illumination, and motion artifacts. The FRET-based indicators are inherently ratiometric, but their dynamic ranges are often relatively small [5]. Several GCaMP-type single FP-based ratiometric Ca^2+^ indicators have been engineered with improved dynamic ranges such as the monomeric GEX-GECO1, GEM-GECO1, and Y-GECOs [8,9].

**Figure 1:**
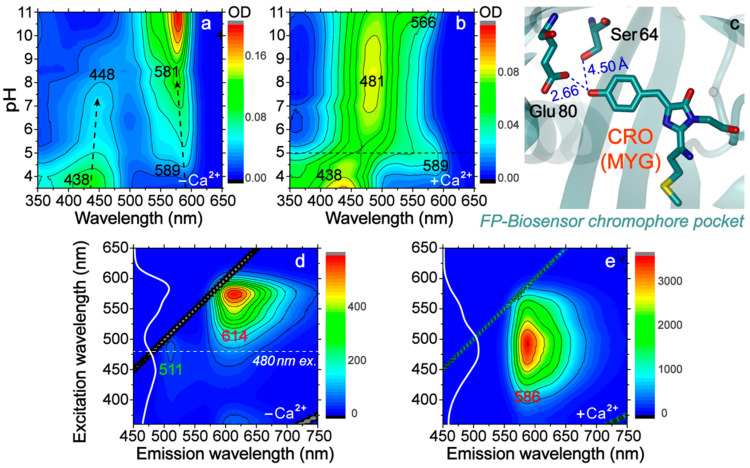
Steady-state electronic spectra of REX-GECO1. The pH-dependent (pH = 3.5–11) UV/Visible absorption spectra of the Ca^2+^ (**a**) free and (**b**) bound biosensors exhibit different patterns. The horizontal dashed line in (**b**) highlights spectral changes across pH = 5. (**c**) Modeled chromophore (CRO) and key residues inside the Ca^2+^-bound REX-GECO1 biosensor. 2D-fluorescence spectra for the Ca^2+^ (**d**) free and (**e**) bound REX-GECO1 in pH = 7 buffer show considerable differences. The ground-state electronic absorption spectra are displayed vertically in white curves.

A novel red fluorescent protein (RFP) based Ca^2+^-biosensor, REX-GECO1, has been engineered and implemented as an intensiometric and excitation-ratiometric indicator with >100 nm Stokes shift and large dynamic range for real-time dynamic Ca^2+^ imaging [10,11]. However, its working mechanism remains unclear with several intriguing questions. For example, at physiological pHs, this biosensor in the Ca^2+^-free state has two absorption bands that correspond to its neutral and anionic forms (see Figure 1a,d and Figure S1b in the SM), while the two bands coalesce into one in the Ca^2+^-bound state (Figure 1b,e and Figure S1b). This unusual behavior distinguishes REX-GECO1 from typical FPs. Another interesting point is that the Ca^2+^-free biosensor is dimly fluorescent with excitation of its neutral form at physiological pHs, while the Ca^2+^-bound state emits strongly with a red-shifted emission peak.

In this contribution, we aimed to delineate the working mechanism of REX-GECO1 and demonstrate its “hidden” capability as a dual-color emission-ratiometric biosensor. Our spectroscopic and computational results indicate that in this biosensor, Ca^2+^ functions as a switch to toggle the excited-state pathways of the chromophore between the ultrafast transition to a dark state and excited-state proton transfer (ESPT) [12]. With this new insight, Ca^2+^-sensing with dual emission wavelengths was achieved in cultured human cells using REX-GECO1, which to our knowledge is the first standalone green-red emission ratiometric Ca^2+^-biosensor to date.

## 2. Results and Discussions

### 2.1. Two-Dimensional Steady-State Electronic Spectroscopy Uncovers Important Amino Acid and Potential Applications

We first performed the pH-dependent absorption measurements of REX-GECO1 to investigate its ground-state electronic property. Both the Ca^2+^-free and bound REX-GECO1 share the same absorption profile, with two peaks at 438 and 589 nm due to the neutral and anionic chromophores in pH < 5 buffer (Figure 1a,b and Figure S1a in the SM). The Ca^2+^-free biosensor largely maintains its absorption profile at high pHs with slight peak shifts (e.g., 448 and 581 nm in pH = 7 buffer), whereas the Ca^2+^-bound state exhibits only one major peak once the pH is raised above 5 (e.g., 481 nm in pH = 7 buffer, see the horizontal dashed line in Figure 1b). This dramatic spectral change must be related to the distinction in chromophore pocket. A modeled chromophore environment based on the crystal structure of the Ca^2+^-bound R-GECO1 (PDB ID: 4I2Y) [13] shows a glutamic acid (Glu80) in close proximity to the phenolic end of the three-residue Met223-Tyr224-Gly225 (MYG) chromophore (CRO, Figure 1c), which is the active site for a photosensory response. The strong interaction formed between Glu80 and CRO in the Ca^2+^-bound biosensor is due to structural changes of the Ca^2+^-binding unit of typical GCaMP biosensors: while wrapped around its binding client, calmodulin can close the β-barrel opening of the FP unit and compress cavity volume surrounding the CRO (Figure 1c) [10,14]. This interaction is expected to be weaker in the Ca^2+^-free biosensor due to a larger cavity size while Glu80 is directed away from the CRO.

Given that Glu has a pK_a_ of ~4.25 for its sidechain carboxylic acid, we surmise that the change of its protonation state can substantially affect the CRO electronic properties. Our quantum chemistry calculations [15] on the effect of Glu (see Experimental methods in Section 3.4 below) show that in comparison to carboxylic acid group, the carboxylate can significantly redshift the energy gap between the highest occupied molecular orbital (HOMO) and lowest unoccupied molecular orbital (LUMO), and lengthen the phenolic OH bond of the CRO (Figure S2 in the SM). This result confirms the significance of an adjacent charged residue in affecting the electronic transition of the photosensory CRO unit, which helps to explain the dramatic spectral change across pH = 5 in the Ca^2+^-bound REX-GECO1 (Figure 1b) and ultrafast spectroscopic features of the biosensor in action (see below).

The biosensor emission properties were investigated through two-dimensional (2D) fluorescence measurements (Figure 1d,e). REX-GECO1 displays similar fluorescence intensity at ~510 and 610 nm (check the color bar) upon 480 nm excitation in the Ca^2+^-free state, while it dominantly emits at ~585 nm in the Ca^2+^-bound state (see Figure S1d and caption in the SM for more details). Since the Ca^2+^-free biosensor absorption band maximum is at 448 nm in pH = 7 buffer (see Figure 1a and Figure S1b) while the 511 nm emission band seems to be best excited at ~480 nm (Figure 1d), it implies that the chromophore subpopulation with a redder absorption peak (than the subpopulation with a bluer absorption peak) is more emissive. Notably, the emission ratio of 610/510 nm after 480 nm excitation exhibits a 48-fold Ca^2+^-dependent change, indicating its potential as an emission-ratiometric indicator. Although the dynamic range is smaller than its excitation ratio (100-fold, 585 nm/480 nm) [10], emission ratios eliminate the signal dependence on stability of the excitation sources, thus offering a more accurate detection. Notably, this attribute only exists for REX-GECO1 biosensor at physiological pHs because the fluorescence at lower pHs shows a similar pattern regardless of Ca^2+^ (see Figure S3 in the SM).

### 2.2. Femtosecond Transient Absorption Signatures Elucidate Distinct Excited-State Pathways for Calcium Sensing

**Figure 2:**
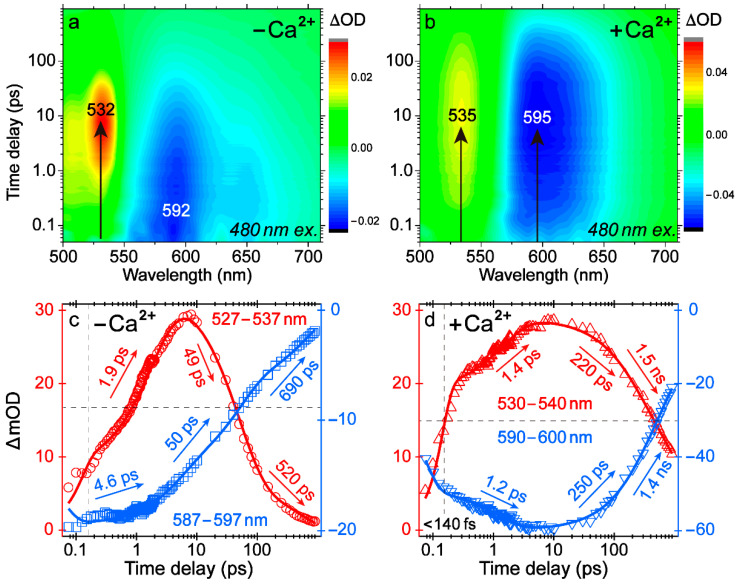
Femtosecond transient absorption (fs-TA) spectroscopy elucidates the excited-state pathways of REX-GECO1. 2D-contour plots for the Ca^2+^ (**a**) free and (**b**) bound biosensors in pH = 7 buffer upon 480 nm excitation show prominent electronic bands that rise and decay within hundreds of picoseconds. Intensity dynamics of representative 10-nm band regions are correspondingly plotted in (**c**,**d**), respectively.

To uncover the excited-state electronic dynamics governing the biosensor emission properties, femtosecond transient absorption (fs-TA) with a 480 nm pump was performed (Figure 2 and Figure S4 in the SM). The Ca^2+^-free biosensor shows a weak and broad stimulated emission (SE) band at 592 nm that decays, while a strong and rather sharp excited-state absorption (ESA) band centered at 532 nm rises on the picosecond (ps) time scale (Figure 2a,c). In contrast, the Ca^2+^-bound REX-GECO1 has a strong SE band at 595 nm and an ESA band at 535 nm, both of which rise and then decay (Figure 2b,d). For the Ca^2+^-free and bound states, SE band above 550 nm is due to a downward transition between the excited (S_1_) and ground (S_0_) states of the deprotonated species, confirmed by the fs-TA spectra with 570 nm excitation, wherein the deprotonated chromophores are predominantly pumped (see Figures S4c and S5 in the SM). Therein, we also displayed the raw fs-TA data traces with two characteristic excitation wavelengths of 480 and 570 nm, highlighting the pure electronic response of the protonated and deprotonated chromophores in the Ca^2+^-bound and free biosensors, respectively.

Since mainly the neutral chromophore of the Ca^2+^-bound REX-GECO1 absorbs at ~480 nm, observing an SE band from the excited-state deprotonated species provides strong evidence that ESPT occurs, leading to a large Stokes shift (>100 nm). Glu80 likely serves as the proton acceptor, similar to Glu160 or Asp160 in LSSmKate FPs [16,17]. The mirror-image pattern of the ESA and SE dynamics infers their common ESPT origin, resulting in the same photoproduct electronic state with upward and downward transitions (Figure 2d) [18]. Notably, the SE and ESA bands exhibit a two-stage rise with a significant portion within the cross-correlation time (<140 fs), which indicates that most of ESPT occurs faster than the instrument response time. This result is corroborated by decay-associated difference spectra (DADS) via global analysis (Figure S6b in the SM) [19], wherein the black and red traces feature an SE band centered at ~520 nm, a signature of the excited protonated species (reminiscent of the similar protonated chromophore emitting at ~510 nm in the Ca^2+^-free biosensor), which decays with ~90 fs and 1.8 ps time constants. These decay processes lead to a rising SE band at ~600 nm as manifested by the evolution-associated difference spectra (EADS) (Figure S6a in the SM), while the retrieved fs and ps components are in accord with the largely matching SE/ESA band rise time constants of <140 fs and 1.2/1.4 ps, respectively (see Figure 2d).

To rationalize these time constants, it was reported that with a nearby Asp, the ESPT rate inside GFP-H148D could be extremely fast (~100 fs) due to the formation of a short hydrogen bond with covalency between CRO and Asp [20,21,22,23]. Based on our calculations (Figure S2 in the SM), interaction with a nearby anionic Glu sidechain elongates the phenolic-OH bond length, thus making the proton in a quasi-stable state (shared between CRO and Glu) and greatly accelerating the ESPT rate (e.g., the retrieved ~90 fs component from global analysis above) upon photoexcitation. Meanwhile, the biosensor inhomogeneous chromophore subpopulations could lead to two distinct ESPT pathways operating on the sub-ps and few ps time scales [18,24].

Regarding the 592 nm SE band in the Ca^2+^-free biosensor, since the deprotonated chromophore can also be excited at 480 nm (Figure 1a and Figure S1b in the SM), its exact origin is unclear. Different time constants appear when comparing the dynamics of this SE band under 480 and 570 nm excitation (see Figure 2c, Figures S5c and S6c in the SM), indicative of some ESPT capability in the former case (see additional discussions in the SM). The shoulder SE band at ~655 nm is likely due to vibronic coupling because it shares similar dynamics with the 592 nm main SE band (Figure S4d in the SM) [25,26]. Importantly, the dramatic difference between ESA and SE band dynamics in Figure 2c (e.g., different time in reaching their peak intensities, a prominent rise versus a slower decay within ~10 ps) indicates that these two electronic bands track different excited-state processes after 480 nm excitation of mainly the protonated chromophore (Figure S1b). Considering that the Ca^2+^-free biosensor emits dim fluorescence [10] with 480 nm excitation (and across the absorption band of the protonated chromophore, see Figure 1d and Figure S1d), a strong and narrow ESA band could be a signature of a dark state (which differs significantly from a bright emissive state of the CRO as shown by direct comparison of the ESA bands in Figure S4c), accumulating with a characteristic 1.9 ps time constant from a chromophore dominant subpopulation inside the Ca^2+^-free REX-GECO1. Moreover, this band is unlikely hot ground state absorption (HGSA) due to its swift rise and lack of blueshift [27,28].

### 2.3. Quantum Calculations and Molecular Dynamics Simulations Suggest the Nature of the Dark State

**Figure 3:**
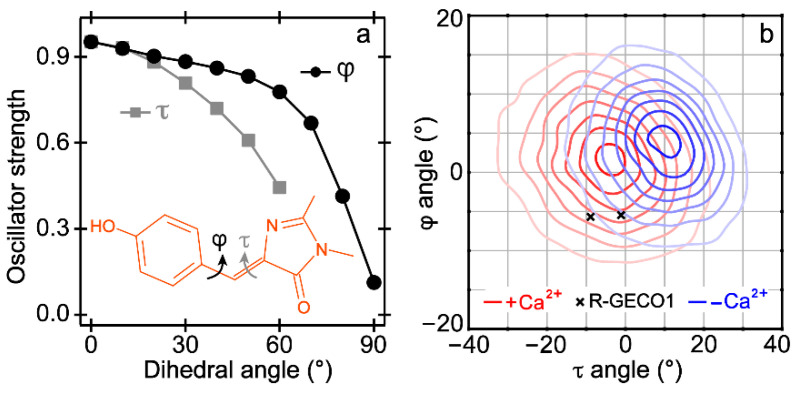
Quantum calculations (**a**) and molecular dynamics simulations (**b**) indicate a more twisted structure of the Ca^2+^ free than bound REX-GECO1 in the excited and ground states. Two key dihedral angles of the chromophore are shown in the (**a**) inset. On the density contour plots in (**b**), angles from the Ca^2+^-bound R-GECO1 (progenitor of REX-GECO1) crystal structure are marked (black crosses).

With a larger cavity volume, the CRO in the Ca^2+^-free biosensor could undergo more twisting motions than the Ca^2+^-bound case. Quantum calculations (see Experimental methods below) show that rotation of two bridge-dihedral angles of the CRO motif in S_1_ (Figure 3a inset) significantly decreases the S_1_→S_0_ transition oscillator strength, supporting the dark nature of this state (Figure 3a). A recent study on the GFP CRO, 4′-hydroxybenzylidene-1,2-dimethyl-imidazolinone (*p*-HBDI), at various temperatures in gas phase suggested the existence of a transient non-fluorescent excited state through the rotation of φ angle [29]. However, the excited HBDI in solution often twists toward 90° to a conical intersection (CI) on the few-ps time scale, resulting in a weak HGSA band that blueshifts due to vibrational relaxation in S_0_ [30,31]. This type of CI is dubious for the CRO in REX-GECO1 due to confinement of surrounding residues in the protein matrix and such a blueshift is absent in the fs-TA spectra (Figure 2a). One special case is the reversibly switchable fluorescent protein [32,33,34] wherein a CI exists, but the Ca^2+^-free REX-GECO1 does not display any photoswitching behavior upon 480 nm excitation, while the ~2 ps rise time of the distinctively narrow ESA band (Figure 2a,c) is much faster than previously reported CRO isomerization time constants in typical photoswitchable proteins [32,35]. Therefore, the dark state we observed in REX-GECO1 is rather special with a unique interplay between the CRO electrostatics and sterics in its local protein environment [18,36]. The exact geometry (torsional angles) of the CRO in this dark excited state requires further investigations with high-level quantum calculations, which should inspire further studies on this versatile biosensor.

Moreover, we suspect that the Ca^2+^-free biosensor is already in a more twisted structure in the ground state than the Ca^2+^-bound case. Due to the lack of crystal structures for either state, we conducted molecular dynamics (MD) simulations on REX-GECO1 (see detailed methods in the Experimental Section 3.5 below). The probability density map (darker color represents greater probability) of the τ and φ angles indicate that the CRO in the Ca^2+^-bound case shows a more coplanar geometry (Figure 3b). Additionally, these two dihedral angles from two chains of the Ca^2+^-bound R-GECO1 crystal structure [13] (a parent protein) fall in the map of Ca^2+^-bound REX-GECO1 (red contours), lending support to our MD-simulation results. With a more non-coplanar structure in the ground state, the CRO of the Ca^2+^-free REX-GECO1 is poised to further twist in the electronic excited state and have a higher chance to search phase space and reach a dark state, as corroborated by the significant drop of oscillator strength as the CRO conjugated ring system twists out of planarity in S_1_ (Figure 3a) as well as the much smaller fluorescence intensity (at the peak wavelength of ~510 nm) of the protonated chromophore inside the Ca^2+^-free biosensor with excitation across its absorption band (ca. 350–490 nm, Figure 1d).

### 2.4. Green-Red Emission-Ratiometric Biosensing Is Achieved in Cell Imaging

**Figure 4:**
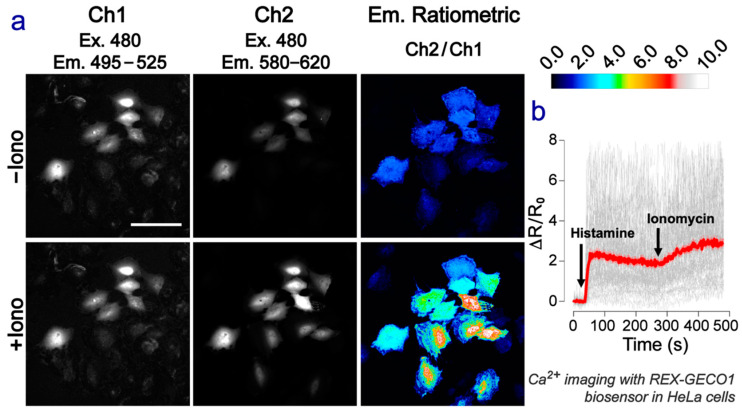
Emission-ratiometric imaging of the REX-GECO1 biosensor. (**a**) Confocal images of HeLa cells expressing REX-GECO1 before (low Ca^2+^) and after (high Ca^2+^) ionomycin treatment. Scale bar = 100 μm. (**b**) Time-lapse Ca^2+^ imaging shown by the red/green fluorescence intensity ratio change (ΔR/R_0_) after 480 nm excitation. Gray lines: single-cell traces. Red line with pink shade: mean ± s.e.m.

To demonstrate the biosensor capability, we expressed REX-GECO1 (*K*_d_ for Ca^2+^ of 240 nM) [10] in cultured human cervical cancer cells (HeLa) and imaged the histamine- and ionomycin-induced increases in Ca^2+^ concentration in the cytosol. As expected on the basis of our spectroscopic measurements with purified proteins, excitation at ~480 nm resulted in green and red emission in the Ca^2+^-free state, which becomes dominantly red in the Ca^2+^-bound state (Figure 1d and Figure 4a). After background subtraction, the average ΔR/R_0_ is ~300% with sufficient signal-to-noise ratios in both green and red channels (70 cells in total, *n* = 3, see the Experimental methods in Section 3.2 below and Figures S7–S10 in the SM for details), where R is the red/green emission ratio (Figure 4b, the red plateau reaching ~3 against the vertical axis at later time). The dynamic range of REX-GECO1 as an emission-ratiometric indicator (Figure 4 and Figure S7 in the SM) outperforms many FRET-based indicators [37,38] and commonly used intensiometric indicators such as jRGECO1a [39] and K-GECO1 [40]. Such a dynamic range also outperforms that derived from the blueshift of the emission peak (F_590_/F_610_, ΔR/R_0_ of 34.4%) when excited at 550 nm (see Figure 1d,e).

Although the Ca^2+^-dependent ratio-fold change is currently lower than GEM-GECO1 (emission ratio) [8] and REX-GECO1 (excitation ratio) [10], additional engineering and optimization can likely produce REX-GECO1 variants with substantially improved dual-color ratiometric emission, an intrinsic property of the CRO as delineated (see above). In contrast to another emission-ratiometric biosensor GEM-GECO1 [8], wherein ESPT is inhibited upon Ca^2+^ binding that leads to the color change from green to blue [14], the ESPT pathway is enabled with Ca^2+^ binding in REX-GECO1. More importantly, the achieved fluorescence is ~100 nm red-shifted relative to GEM-GECO1. However, the REX-GECO1 red-to-green emission-ratiometric method currently suffers from higher noise relative to the excitation-ratiometric method [10] due to the intrinsically dim emission at 510 nm when excited at 480 nm (see Figure 1d, Figures S1d and S8a).

## 3. Materials and Methods

### 3.1. Protein Purification, pH-Dependent Absorption, and 2D-Fluorescence Measurements

To purify the REX-GECO1 proteins, pTorPE-REX-GECO1 plasmids were transformed into DH10B *E. coli* competent cells through electroporation. Cells were placed on a Luria broth (LB) agar plate supplemented with 1× ampicillin and 0.002% (wt/vol) arabinose for overnight culture at 37 °C [8,14,24]. On the next day, single colonies were picked from liquid culture in 500 mL LB media containing 1× ampicillin and 0.002% (wt/vol) arabinose. After 48 h, cells expressing REX-GECO1 were collected by centrifugation, resuspended in 30 mM Tris-buffered saline (pH = 7.4), and lysed by sonication. The supernatant was separated by centrifugation at 14,000× *g* for 30 min. Proteins were extracted and purified by Ni-NTA affinity chromatography (MCLAB).

The in vitro buffer solutions with pH values ranging from 3.5 to 11 (with a step of 0.5) were prepared, containing 30 mM trisodium citrate, 30 mM borax, and 10 mM EGTA (Ca^2+^-free sample) or Ca^2+^-EGTA (Ca^2+^-bound sample). REX-GECO1 proteins were diluted into the buffers with specific pH to achieve μM concentration [24]. The protein biosensor absorption spectra were then measured using the UV/Visible spectrophotometer (Beckman DU-800, see Figure 1 and Figure S1; as well as Thermo Scientific Evolution 201).

We performed the 2D-fluorescence measurements via a spectrofluorophotometer (SHIMADZU RF-6000). In particular, we utilized the 3D scan feature and set the excitation wavelength from 360 to 650 nm (with 5.0 nm interval), and emission wavelength from 370 to 800 nm (with 1.0 nm interval). The scan speed was set at 2000 nm/min. A quartz cuvette with 5 mm pathlength was used. The protein sample concentration was diluted to ~10 μM. For REX-GECO1 in the pH = 7 buffer, the excitation and emission slit width were set at 3.0 nm with low sensitivity. For the sample in the pH = 4 buffer, the excitation slit width was set at 5.0 nm and emission slit width was set at 3.0 nm with low sensitivity.

### 3.2. HeLa Cell Culture and Imaging

HeLa cells were cultured, transfected, and imaged similarly as described previously [10]. Briefly, plasmids of CMV-REX-GECO1 (Addgene #61246) were prepared from *E. coli* DH10B cells using GeneJET Plasmid Miniprep Kit (Thermo Scientific). For mammalian cell transfection, 2 μg of plasmid DNA was transfected to HeLa cells using polyethylenimine (PEI, linear MW 25K, Polysciences) 24 h after being seeded onto imaging cover glasses. 5 h after incubation with the transfection mixture, the medium was replaced with pre-warmed fresh F12/DMEM (Thermo Fisher Scientific) containing 10% fetal bovine serum and 1% penicillin-streptomycin-amphotericin where the cells were incubated at 37 °C for an additional 19 h before imaging.

To image the histamine- and ionomycin-induced Ca^2+^ signaling, the transfected cells on the cover glass were transferred to an Attofluor cell chamber (Invitrogen) and with imaging buffer containing 107 mM NaCl, 7.2 mM KCl, 1.2 mM MgCl_2_, 11.5 mM glucose, 20 mM HEPES-NaOH (pH 7.2), and 1 mM CaCl_2_. Histamine and ionomycin were added as indicated in Figure 4b and Figure S8 in the SM. Images were acquired on a Leica TCS SP8 microscope equipped with a Fluotar VISIR 25×/0.95 water immersion objective lens. A white-light laser with an acousto-optic tunable filter (AOTF) and acousto-optic beam splitter (AOBS) was used for the excitation line selection, switching between 480 nm (4% emission power) and 550 nm (2% emission power) sequentially on a line-by-by basis. Emission light was dispersed using a Leica prism-based detector system for wavelength selection, with 495–525 nm (green) channel and 580–620 nm (red) channel collected simultaneously by two PMT detectors. The former detection channel largely avoids the bleed-through from the strong red emission peak so the weak green emission peak can be tracked. The recording lasted for ~8 min with images acquired at 16-bit every 2 s with a pixel dwell time of 3.26 µsec. Since the spatial resolution is not critical for calcium imaging in the cytosol, the physical pinhole diameter was set to 600 µm (12.2 airy units for 510 nm and 10.4 airy units for 600 nm) to maximize photon collection by appropriate oversampling (pixel size = 1.21 µm/pixel). To obtain images (see Figure 4a in main text and Figure S7 in the SM) at the representative high resolution, the laser power at 480 nm and 550 nm was increased to 20% and 7%, respectively, while the pixel dwell time was increased to 19.5 µsec and the pixel size was decreased to 0.158 µm.

We note that 480 nm excitation of the REX-GECO1 indicator could generate detectable fluorescence signals from both red and green channels with the available optical filters (see above, captured as separate images in Figure 4a and Figure S7 in the SM). With excitation wavelengths shorter than 450 nm, green emission for the cell resting state (~150 nM Ca^2+^) was too weak to be detected in the microscope (see 2D-fluorescence map in Figure 1d for corroboration).

After ~40 s of initial recording of the resting state, histamine was added to reach a final concentration of 100 μM to induce the Ca^2+^ oscillation (note that REX-GECO1 has a binding affinity *K*_d_ of 240 nM) [10]. Moreover, ionomycin (2.5 μM added after ~280 s) permeabilizes the cell plasma membrane to Ca^2+^ and induces the store-operated calcium entry (SOCE), which is supposed to saturate the REX-GECO1 biosensors with Ca^2+^ [40].

To compare the fluorescence intensity from HeLa cells expressing REX-GECO1 under different excitation and emission conditions, a manual λ-scan was performed on the Leica TCS SP8 system with a HC PL APO CS2 100×/1.40 oil immersion objective lens. Excitation beams were selected using AOTF and AOBS from a white-light laser and kept at 6% emission power, switching between 480 and 550 nm sequentially on a line-by-by basis. Emission light was dispersed using a Leica prism-based detector system. Signal was recorded with the same PMT kept at constant gain mode from 30 nm bandwidth collection windows, of which the central wavelengths were indicated in Figure S9 in the SM. The collection window was moved across the spectrum with a 20 nm increment.

All images acquired for analysis were subjected to a mean filter (radius = 2 pixels) in ImageJ program, following which the background subtraction, region of interest (ROI) selection, and ratio calculation were performed with a custom Python script. The mean filtering step smoothens the pixel-to-pixel fluctuations and obtains ratio values similar to the ROI-based method (see Figure S10 in the SM). The pseudo-colored images and movies were generated with ImageJ while other figures were plotted with R and GraphPad Prism 7. For corroboration, a representative time-lapse (~500 s) movie of the emission- and excitation-ratiometric imaging experiments of HeLa cells expressing the REX-GECO1 biosensor before (low Ca^2+^) and after (high Ca^2+^) histamine and ionomycin treatment was provided as a supplementary online movie (Video S1).

Notably, the magnitude of fluorescence signal change in cultured cells should correlate to, but not directly compare to that in vitro according to all the previous FP-based Ca^2+^ indicators [7,8]. Besides the aforementioned less contrast of Ca^2+^ level in cells (from high nM to low μM) than that in buffer (from zero to mM), the protein indicators expressed in cells are in a more complex matrix where indicators do not work that well as they do in buffer. As a result, there are visual variations of fluorescence signals from individual cells in response to the ionomycin-induced Ca^2+^ concentration change (see individual-cell traces and the mean for all ROIs in Figure 4b and Figure S8 in the SM). For the total of 70 cells examined and pooled from three replications, an average ratio change of ~300% (ΔR/R_0_, where R_0_ is the initial ratio) was achieved after background subtraction, while the signal-to-noise ratio (SNR) in the green channel is above 3 with the mean filtering (Figure S10 in the SM). These imaging data substantiate the functionality of REX-GECO1 as a single FP-based emission-ratiometric Ca^2+^ indicator (with room for further improvement) to be the second-generation genetically encoded calcium ion indicators (GECIs) with redder emission wavelengths that could greatly benefit bioimaging applications [40].

### 3.3. Femtosecond Transient Absorption Spectroscopy

The femtosecond transient absorption (fs-TA) setup in our lab has been reported before [12,41]. In brief, a 1 kHz-repetition-rate Ti:sapphire regenerative amplifier (Legend Elite-USP-1K-HE, Coherent, Inc., Santa Clara, CA, USA) produces fundamental pulses with ~800 nm center wavelength, ~35 fs duration, and ~3.7 W average power. The fs 480 or 570 nm photoexcitation pulse was converted from the fundamental output pulse via a home-built two-stage noncollinear optical parametric amplifier (NOPA), followed by passage through a chirped mirror pair (DCM-12, 400–700 nm, Laser Quantum, Inc., Stockport, UK) for temporal compression [42]. The probe pulse as supercontinuum white light was generated by focusing a portion of the fundamental laser output onto a 2-mm-thick quartz cell filled with deionized water, then compressed via a chirped mirror pair (DCM-9, 450–950 nm, Laser Quantum, Inc., Stockport, UK) in the time domain. The actinic pump power was set at ~0.2 mW before the phase-locked optical chopper (Newport/New Focus 3501). The pump and probe pulses were focused on the sample solution housed in a 1-mm-thick quartz flow cell. After the sample, the probe beam was collimated and focused into a spectrograph (IsoPlane SCT-320, Princeton Instruments, Inc., Trenton, NJ, USA) with a reflective grating (300 grooves/mm, 300 nm blaze wavelength), and then imaged on a front-illuminated CCD array camera (PIXIS:100F, Princeton Instruments, Inc.) with Lumogen UV coating. For fs-TA spectral data collection, the absorption OD of the protein sample solution was tuned to ~0.25 per mm at the excitation wavelength (480 or 570 nm).

### 3.4. Quantum Mechanical Calculations

To investigate the electrostatic effect of glutamic acid (Glu) on the REX-GECO1 chromophore (CRO), we performed quantum calculations of three scenarios: CRO alone, neutral Glu with CRO, and anionic Glu with CRO using Gaussian 16 software [15]. The neutral/protonated CRO was constructed as 4′-hydroxybenzylidene-1,2-dimethyl-imidazolinone (*p*-HBDI) to decrease the calculation cost and avoid complication effects of less crucial long sidechains to calculation results. The molecular structures were optimized with density functional theory (DFT), RB3LYP functional and 6-311G+(d,p) basis sets on the electronic ground state (S_0_). The HOMO and LUMO energies (see Figure S2 in the SM) can be correlated with the vertical transition energy gap that corresponds to the experimentally measured electronic absorption peak (see Figure 1 and Figure S1 in the SM).

To explore the possible origin of the dark state as a twisted state in the Ca^2+^-free REX-GECO1, we separately twisted two exocyclic dihedral angles (τ angle closer to the imidazolinone ring, and φ angle closer to the phenolic ring as illustrated in Figure 3a) of the CRO with a 10° step from 0° to 90° while keeping the other angle unchanged. The chromophore structures were optimized in the first singlet excited state (S_1_) with time-dependent (TD)-DFT method, RB3LYP functional, and 6-31G+(d,p) basis sets. The excited state geometrical optimization cannot converge with τ angle beyond 60° likely due to severe disruption of the chromophore conjugation structure, hence the truncated calculation result plot (gray trace in Figure 3a). The oscillator strength between S_1_ and S_0_ states at the S_1_ optimized geometry was retrieved from the output (.log) file after each dihedral-angle-specific calculation. We note that the transition oscillator strength between S_1_ and S_0_ states at the excited-state optimized structures with fixed dihedral angles of the methine-bridge bonds displays a monotonous decrease with a more twisted chromophore.

### 3.5. Molecular Dynamics Simulations

We performed molecular dynamics (MD) simulations on the REX-GECO1 biosensor to determine its structural differences between the Ca^2+^ bound and free states. REX-GECO1 has no available crystal structure, so we relied on homology modelling to generate initial structures. REX-GECO1 and other GCaMP2-derived proteins consist of a circularly permutated fluorescent protein along with a calmodulin (CaM, the calcium-binding unit) and an M13 peptide (the CaM-binding region of chicken myosin light-chain kinase) [8,14,43,44]. Calcium binding induces a reorientation of the CaM and M13 regions, which affects the protein’s fluorescence properties. R-GECO1 is a close analogue to REX-GECO1, and it has a crystal structure available for its Ca^2+^-bound state (PDB ID: 4I2Y) [13], making it our choice for target structure of the Ca^2+^-bound REX-GECO1. The closest available structure for a Ca^2+^-free protein is GCaMP2 (PDB ID: 3EKJ) [44], which has a much lower sequence similarity to REX-GECO1 relative to R-GECO1 (52 vs. 97%); it does, however, capture the location of the CaM unit in this state. We thus constructed the Ca^2+^-free REX-GECO1 using the fluorescent protein unit of 4I2Y and a separate Ca^2+^-free CaM structure (PDB ID: 1CFD) [45]. The relative orientation of these structural units within the biosensor protein chimera was determined using 3EKJ as a reference [14,44].

Initial structures were generated using MODELLER. Five structures in both the Ca^2+^-free and bound states were selected for MD simulations, which used the July 2017 version of the CHARMM36 all-atom force field. Previous literature provided the parameters for the protonated tyrosine-derived chromophore *p*-HBDI [14,46,47]. The systems were solvated in a 14 Å box of TIP3P water molecules, then neutralized and ionized with sodium and chloride ions to an ionic concentration of 0.1 M. System preparation was conducted through the psfgen, solvate, and autoionize plugins in the molecular graphics program VMD [48].

The MD simulations were performed in NAMD 2.13, an open-source MD code designed for high-performance simulation of large biomolecules. All systems were energy minimized for 50,000 steps using the NAMD default conjugate gradient algorithm. Following minimization, the systems were equilibrated for 1 ns under *NPT* conditions, followed by a 1 ns equilibration using the *NVT* ensemble. Backbone atoms were harmonically restrained with a 10 kcal·mol^−1^·Å^−1^ force constant during the *NPT* equilibration. These restraints were gradually released by reducing the force constant during the *NVT* equilibration; force constants of 10, 5, 1, and 0 kcal·mol^−1^·Å^−1^ were applied sequentially at 250 ps intervals. Finally, five parallel runs of 50 ns *NVT* production simulations generated the data that were used in the ensuing analysis (e.g., Figure 3b). Since an aggregate of five simulation trajectories with different starting configurations was used, resulting in 250 ns in simulation time for each biosensor state (i.e., Ca^2+^-free or bound REX-GECO1), the conformational phase space and energy landscape of the embedded chromophore were expected to be sufficiently sampled (better than a longer simulation with a single starting configuration, which may lead to a possible trap or unrealistic drift) [49].

All equilibration and production simulations used a 2 fs time step. Bonded forces and short-range non-bonded forces were evaluated at every time step, and long-range electrostatics at every other time step. All bonds to hydrogen atoms were treated as rigid bonds using ShakeH [14]. Non-bonded interactions were treated with a 12 Å cutoff, with a switching function used between 10 and 12 Å. Scaled 1–4 exclusions were applied to exclude nearby atoms from non-bonded interactions, with the electrostatics of 1–4 pairs scaled by 1.0. Particle Mesh Ewald (PME) with a 1.0 Å grid spacing was used for electrostatic interactions. Periodic boundary conditions (PBC) were used at the edges.

A constant temperature of 300 K (27 °C) was set in all simulations through Langevin dynamics on all non-hydrogen atoms, using a 5.0 ps^−1^ damping constant. During the *NPT* equilibration, a Nosé-Hoover Langevin piston maintained a target pressure of 1.01325 bar (1 atm); this piston had an oscillation period of 100 fs and a 50 fs damping time scale.

Trajectories were reduced in size for analysis (by removing solvent) using Pytraj and VMD after the simulations completed. We used MDAnalysis, NumPy, Matplotlib, and Seaborn (all open-source codes) to extract and visualize information from these trajectories.

The evenly spaced frames from each of the simulations at 20 ps intervals for a total of 2500 frames per simulation were used to explore the dihedral angles between the protein chromophore’s two rings (see Figure 3a inset). The probability density of the resultant angles was retrieved with kernel density estimation using a Gaussian kernel and a bandwidth determined by Scott’s rule, which visually represent a scatter plot of the exact angles sampled. The plot shows this estimate for each protein state (i.e., the Ca^2+^-free and bound REX-GECO1 biosensors), with darker contours representing higher probability densities for the τ angles between ca. −30° and 30°, and φ angles between ca. −10° and 15° (see Figure 3b).

## 4. Conclusions

In summary, we revealed the working mechanism of REX-GECO1 with a series of spectroscopic and calculation methods. The excited-state pathway inside the biosensor can be switched between a twisted-chromophore dark state and barrierless ESPT before and after Ca^2+^-binding. The importance of a nearby Glu80 in the chromophore vicinity was highlighted. For the first time, the green/red dual-color Ca^2+^ sensing was achieved in human cells with a single FP-based biosensor, which offers a new design strategy for Ca^2+^ biosensors. Moving forward, REX-GECO1 can be engineered to further improve its dynamic range as an emission-ratiometric reporter. This could be achieved by directed evolution with strategic mutations [7,31,50] to enhance red emission from the Ca^2+^-bound state, while reducing the CRO cavity volume with more hydrophobic residue sidechains to trap the Ca^2+^-free biosensor in an ESPT-incapable, brighter green state.

## Data Availability

Not applicable.

## Supplementary Materials

The following are available online at https://www.mdpi.com/1422-0067/22/1/445/s1, Figure S1: Steady-state electronic absorption spectra of the Ca^2+^-free and bound REX-GECO1 in aqueous buffer solutions at pH = 4, 7, and 11, and the emission spectra in aqueous buffer at pH = 7 after 480 nm excitation, Figure S2: Calculated energies and the chromophore phenolic O–H bond lengths for HBDI without/with an adjacent neutral or anionic Glu residue, Figure S3: 2D-fluorescence spectra for the Ca^2+^-free and bound REX-GECO1 in pH = 4 buffer, Figure S4: Fs-TA spectra and dynamics of the Ca^2+^-free and bound REX-GECO1 with 480 nm excitation at various time delays in pH = 7 buffer, Figure S5: 2D-contour plots of the fs-TA spectra of the Ca^2+^-free and bound REX-GECO1 after 570 nm excitation in pH = 7 buffer, Figure S6: Global analysis of the fs-TA spectra of the Ca^2+^-bound/free REX-GECO1 after 480/570 nm excitation in pH = 7 buffer, Figure S7: Contrasting plots of the emission- and excitation-ratiometric imaging data of the REX-GECO1 biosensor in HeLa cells, Figure S8: Fluorescence signal and change at each individual imaging channel, Figure S9: λ-scan performed on HeLa cells expressing REX-GECO1 biosensor, Figure S10: λ-scan imaging data analysis with the mean filtering on HeLa cells expressing REX-GECO1 after 480 or 550 nm excitation, Video S1: A representative time-lapse movie of the emission- and excitation-ratiometric imaging experiments of HeLa cells expressing REX-GECO1 before (low Ca^2+^) and after (high Ca^2+^) histamine and ionomycin treatment within ~500 s.

## Abbreviations

GFP	green fluorescent protein
FRET	Förster resonance energy transfer
RFP	red fluorescent protein
GECO	genetically encoded calcium indicator for optical imaging
GECI	genetically encoded calcium indicator
ESPT	excited-state proton transfer
HOMO	highest occupied molecular orbital
LUMO	lowest unoccupied molecular orbital
fs-TA	femtosecond transient absorption
ESA	excited-state absorption
SE	stimulated emission
HGSA	hot ground state absorption
DADS	decay-associated difference spectra
EADS	evolution-associated difference spectra
*p*-HBDI	4′-hydroxybenzylidene-1,2-dimethyl-imidazolinone
DFT	density functional theory
TD-DFT	time-dependent DFT
MD	molecular dynamics
