# Supplementary material for: Switching between Ultrafast Pathways Enables a Green-Red Emission Ratiometric Fluorescent-Protein-Based Ca2+ Biosensor
Source: Int J Mol Sci. 2021 Jan 5;22(1):445. doi: 10.3390/ijms22010445 (PMC7794744; doi:10.3390/ijms22010445)
Supplement: Supplementary file 1 [file ijms-22-00445-s001.zip › IJMS_REX_SM_rev_CF1_NOHighlights.pdf]

# Supplementary Material (SM) for

# Switching Between Ultrafast Pathways Enables a Green-Red Emission Ratiometric Fluorescent-Protein-Based $Ca^{2+}$ Biosensor

*Longteng Tang,[†] Yufeng Zhao,[§] Shuce Zhang,[§] Nikita D. Rozanov,[†] Liangdong Zhu,[†] Jiahui Wu,[§] Robert E. Campbell,[§,⊥] and Chong Fang[†]**

[†]Department of Chemistry, Oregon State University, 153 Gilbert Hall, Corvallis, Oregon 97331, United States

[§]Department of Chemistry, University of Alberta, Edmonton, Alberta T6G 2G2, Canada

[⊥]Department of Chemistry, Graduate School of Science, The University of Tokyo, 7-3-1 Hongo, Bunkyo-ku, Tokyo 113-0033, Japan

**Corresponding Author**

*E-mail: Chong.Fang@oregonstate.edu

## Table of Contents

Page number

**SM Figures** **S3 – 17**

**Figure S1.** Steady-state electronic absorption spectroscopy of the $Ca^{2+}$-free and bound REX-GECO1 in aqueous buffer solutions at pH=4, 7, 11, and the emission spectra in aqueous buffer at pH=7 after 480 nm excitation S3–4

**Figure S2.** Calculated energies and the chromophore phenolic O–H bond lengths for HBDI without/with an adjacent neutral or anionic Glu residue S5

**Figure S3.** 2D-fluorescence spectra for the $Ca^{2+}$-free and bound REX-GECO1 in pH=4 buffer S6

**Figure S4.** Fs-TA spectra of the $Ca^{2+}$-free and bound REX-GECO1 with 480 nm excitation at various time delays in pH=7 buffer S7–8

**Figure S5.** 2D-contour plots of the fs-TA spectra of the $Ca^{2+}$-free and bound REX-GECO1 after 570 nm excitation in pH=7 buffer S9–10

**Figure S6.** Global analysis of the fs-TA spectra of the $Ca^{2+}$-bound/free REX-GECO1 after 480/570 nm excitation in pH=7 buffer S11–12

**Figure S7.** Contrasting plots of the emission- and excitation-ratiometric imaging data of the REX-GECO1 biosensor in HeLa cells S13

**Figure S8.** Fluorescence signal and change at individual imaging channels S14–15

**Figure S9.** λ-scan performed on HeLa cells expressing REX-GECO1 biosensor S16

**Figure S10.** λ-scan imaging data analysis with the mean filtering on HeLa cells expressing REX-GECO1 after 480 or 550 nm excitation S17

*Additional* ***discussion*** *follows each figure caption.*

**SM References** **S18 – 19**

*An additional* ***Video S1*** *(a representative time-lapse movie of the emission- and excitation-ratiometric imaging experiments of HeLa cells expressing REX-GECO1 before (low $Ca^{2+}$) and after (high $Ca^{2+}$) histamine and ionomycin treatment within ~500 s) that accompanies the paper is available online.*

**SM Figures**

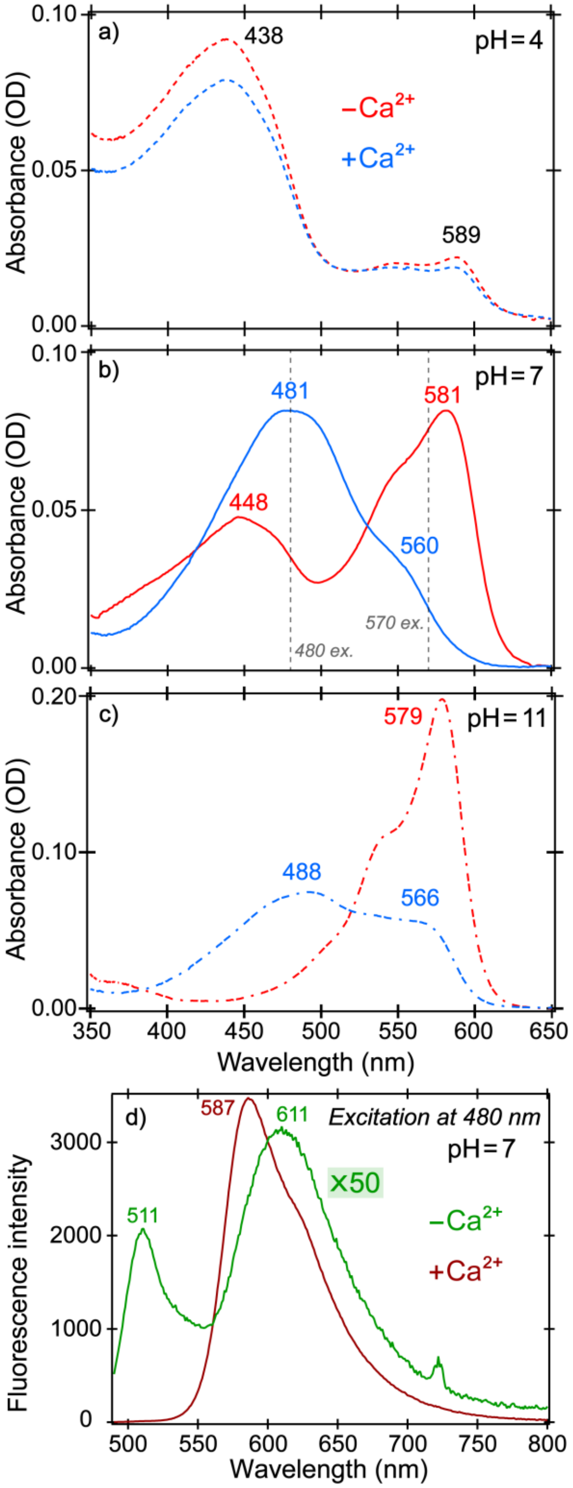


**Figure S1.** Steady-state electronic absorption spectra of the $Ca^{2+}$-free (red) and bound (blue) REX-GECO1 in aqueous buffer solutions at pH = (a) 4, (b) 7, and (c) 11. The protein

sample concentration used for these measurements was ~5 μM. Prominent peak locations are denoted. Vertical dashed lines in (b) denote the photoexcitation pulses at 480 and 570 nm for time-resolved electronic spectroscopic measurements (i.e., fs-TA with higher concentration of protein biosensor samples, see Experimental methods in main text).[1] In panel d, the steady-state emission spectra of the $Ca^{2+}$-free (green) and bound (red) REX-GECO1 in aqueous buffer at pH=7 after 480 nm excitation are shown. The $Ca^{2+}$-free biosensor spectrum is multiplied by 50 to directly compare with the much stronger fluorescence intensity profile of the $Ca^{2+}$-bound biosensor (also see the colorbars in Figure 1d,e in main text).

Notably, upon 480 nm excitation, the $Ca^{2+}$-free REX-GECO1 in pH=7 buffer solution exhibits two prominent emission peaks at ~511 and 611 nm (Figures 1d and S1d), with the former peak (weaker intensity, fwhm≈1100 $cm^{-1}$) distinctively narrower than the latter peak (stronger intensity, fwhm≈2250 $cm^{-1}$). Since the corresponding absorption spectrum shows two main peaks at ~448 and 581 nm from the protonated and deprotonated chromophores, respectively (Figures 1a and S1b), the fluorescence peaks at 511 and 611 nm arise from the photoexcited chromophores with different protonation states as well, while the characteristic narrow spectral width of the 511 nm peak indicates more homogeneity (narrower distribution of the emissive-state configurations with the peak absorption at ~480 nm, see 2D-fluorescence map in Figure 1d) of protonated chromophore. In contrast, the $Ca^{2+}$-bound REX-GECO1 in pH=7 buffer solution displays one dominant emission peak at ~587 nm (fwhm≈1950 $cm^{-1}$) with a small shoulder peak at ~630 nm on the red side, which could arise from vibronic coupling or ground-state inhomogeneous populations of the protonated chromophore inside the $Ca^{2+}$-bound biosensor (main text).

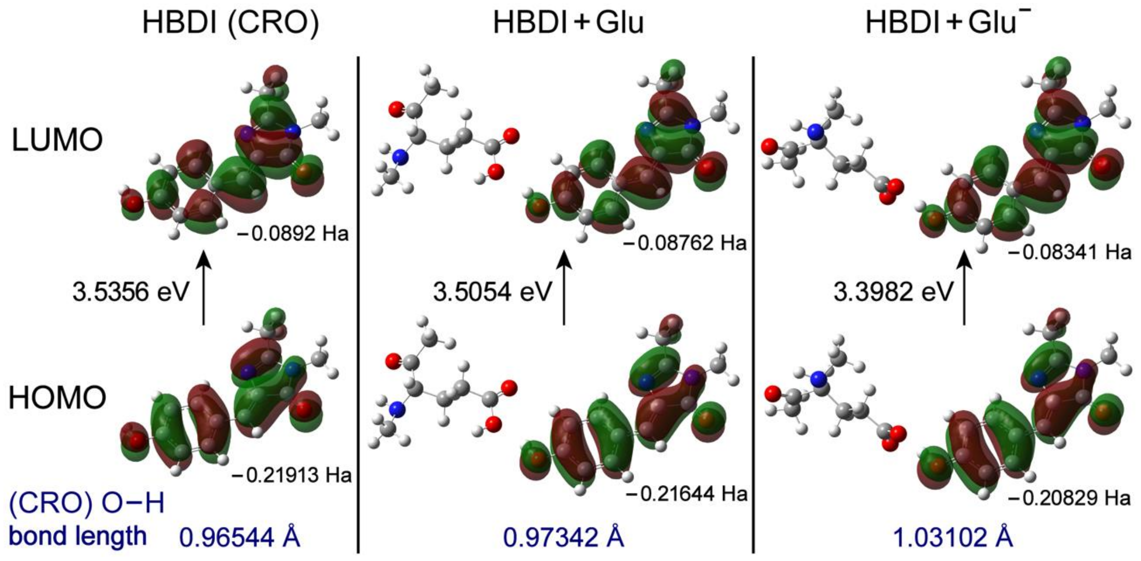


**Figure S2.** Calculated HOMO/LUMO energies and the chromophore phenolic end O–H bond lengths for HBDI (left), HBDI with an adjacent neutral Glu residue (middle), and HBDI with an adjacent anionic Glu residue (right column). The electron density distribution of the MOs is represented by semi-transparent isosurface with negative and positive phases shown in green and red, respectively. The vertical absorption energy gap in eV unit is denoted.[2] Note: HBDI chromophore remains protonated in these calculations.

This systematic comparison confirms that the protonation state change of a nearby Glu residue can shift the electronic absorption peak of the protonated chromophore, as observed for the $Ca^{2+}$-free (438/448 nm at pH=4/7 in Figure S1a/b) and $Ca^{2+}$-bound (438/481/488 nm at pH=4/7/11 in Figure S1a/b/c) REX-GECO1. A smaller redshift magnitude in the $Ca^{2+}$-free biosensor is in accord with a larger distance between CRO and Glu than the $Ca^{2+}$-bound case. Also, the still prominent 488 nm absorption peak of the $Ca^{2+}$-bound biosensor in pH=11 buffer (Figure S1c) attests to a more rigid and compact CRO environment that further stabilizes a protonated chromophore with an adjacent carboxylate from Glu80.

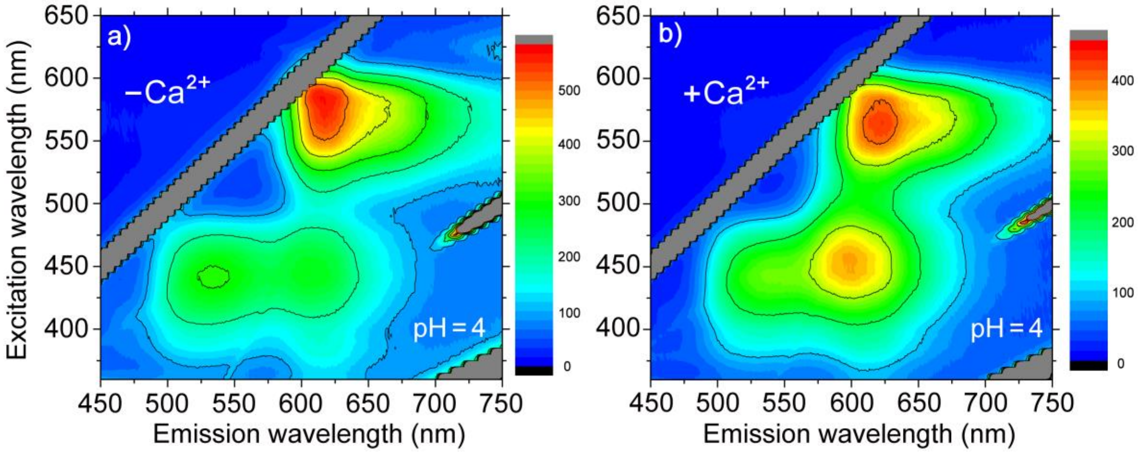


**Figure S3.** 2D-fluorescence spectra for the $Ca^{2+}$ (a) free and (b) bound REX-GECO1 in pH=4 buffer solution. The protein biosensor samples became cloudy at this acidic condition on the minutes time scale, while unstable proteins lead to low fluorescence emission photon counts (see colorbars).

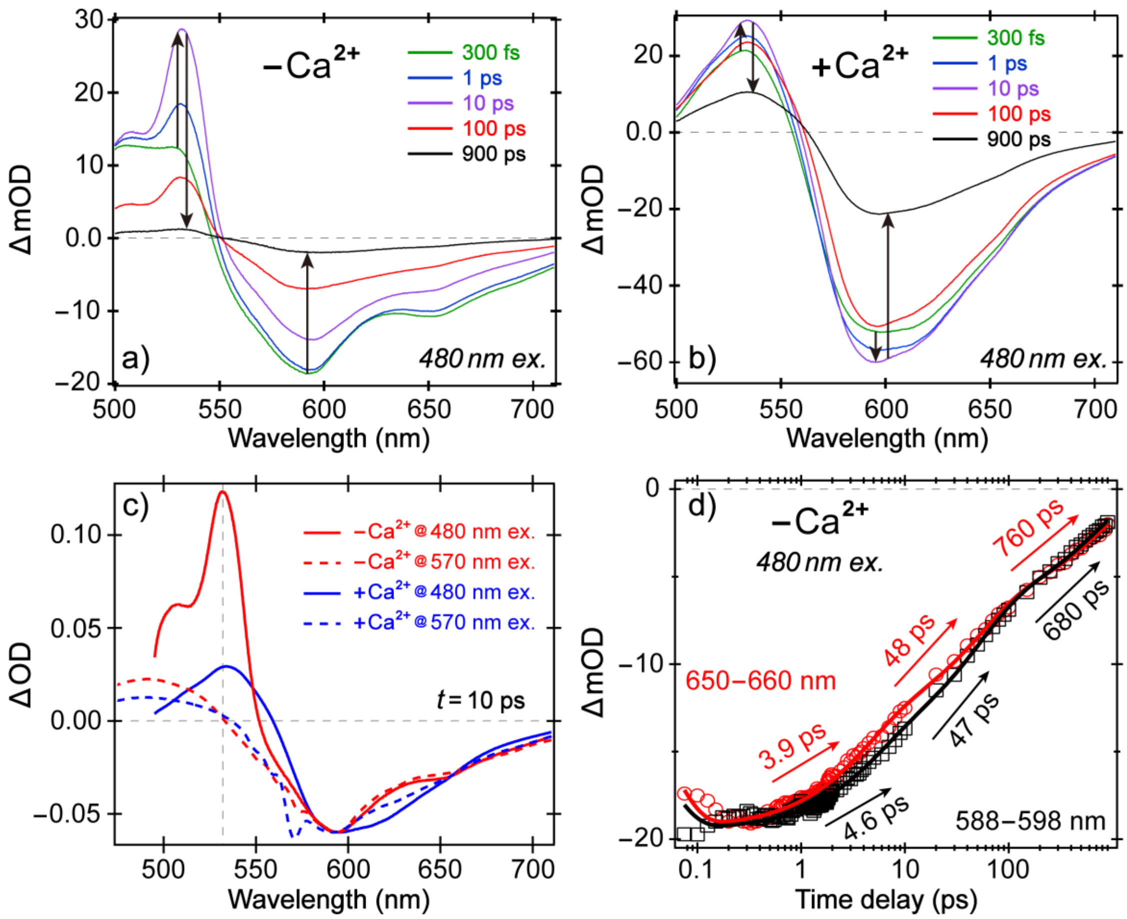


**Figure S4.** Fs-TA spectra of the $Ca^{2+}$ (a) free and (b) bound REX-GECO1 with 480 nm excitation at various time delays (color-coded traces) in pH=7 buffer solution. Key spectral evolution is highlighted by vertical arrows. (c) TA spectra of the $Ca^{2+}$-free and bound REX-GECO1 at 10 ps time delay after the actinic pump with different excitation wavelengths. All the spectra were scaled to match the stimulated emission (SE) peak of the $Ca^{2+}$-bound REX-GECO1 after 480 nm excitation (blue solid trace). Significant differences in the excited-state absorption (ESA) region below ~550 nm are apparent. (d) The scaled intensity dynamics of two SE regions (10-nm width, red and black) of the $Ca^{2+}$-free REX-GECO1 after 480 nm excitation, overlaid with the least-squares fits in solid curves. The retrieved time constants are listed by the corresponding arrows. Time axis is in the logarithmic scale.

Notably, the sharp ESA feature below 550 nm for the $Ca^{2+}$-free REX-GECO1 upon 480 nm excitation (Figure S4a,c) could be characteristic of an excited dark state via chromophore twisting motions (see main text). In particular, this ESA band (apparent in Figure 2a as well) is unlikely to be affected by the ground-state bleaching (GSB) band of the protonated chromophore for the following reasons. First, the 532 nm ESA band is narrow and away from the ~448 nm absorption peak of the protonated chromophore (Figure S1b). Second, the 532 nm ESA band is much stronger (~2 times) than the SE band on the red side (Figure S4c). With 570 nm excitation, the GSB band of the deprotonated chromophore does not show up in the observed SE band at ~593 nm (Figure S5a). Since the protonated chromophore absorbing at ~448 nm has a lower OD than the deprotonated chromophore absorbing at ~581 nm in pH=7 buffer solution (Figure S1b), we conclude that the photoinduced GSB band is likely much weaker than the sharp and strong ESA band at 532 nm (with its largely isolated and distinct dynamics displayed in Figure 2c, red trace).

Moreover, global analysis[3,4] of fs-TA spectra of the $Ca^{2+}$-free REX-GECO1 following 480 nm excitation indicates a weak SE band around 500 nm that is consistent with the ~510 nm fluorescence peak from the excited protonated chromophore (see Figure 1d with excitation wavelength below ~490 nm; note the deprotonated chromophore emission that is redder than ~600 nm). However, due to the excitation of some mixed protonation states of the chromophore in this case (Figure S1b) with overlapped electronic features that hinder detailed analysis of the underlying species, we preferably performed the global analysis of two other experimental conditions tracking the largely pure electronic dynamics for the $Ca^{2+}$-free and bound biosensors with deprotonated and protonated chromophores following 570 and 480 nm excitation in Figure S6c,d and a,b, respectively (see below).

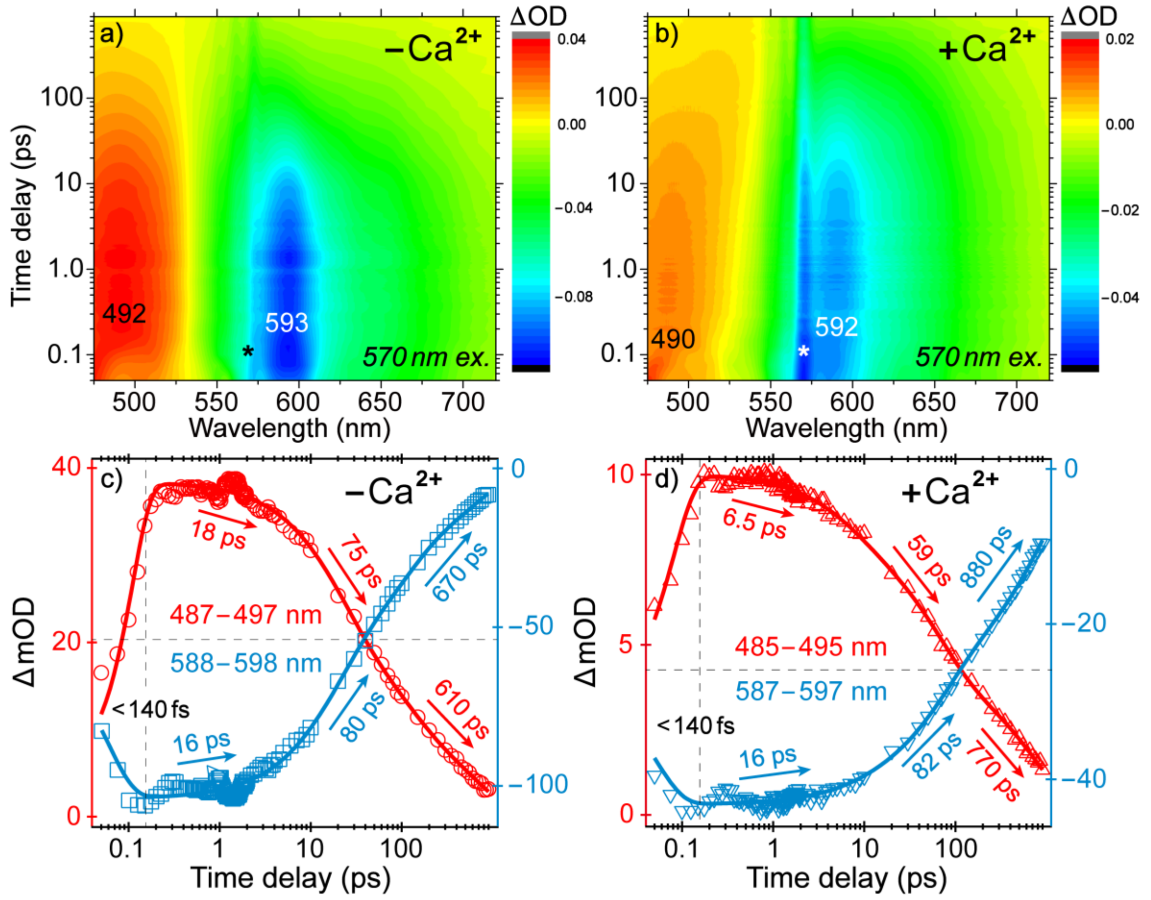


**Figure S5.** 2D-contour plots of the fs-TA spectra of the $Ca^{2+}$ (a) free and (b) bound biosensors after 570 nm excitation in pH=7 buffer solution. Time axis is in the logarithmic scale. The asterisks show scattering from the excitation pulse. The corresponding intensity dynamics of representative spectral regions (10-nm width) are displayed in (c) and (d), respectively, the color-coded data points overlaid with least-squares fits in solid traces. Characteristic time constants retrieved from the fits are shown by their associated arrows. Dashed lines highlight the "mirror symmetry" of the dynamics traces of the bluer ESA band (red, left axis) and redder SE band (cyan, right axis), indicative of their origin from the same electronic excited state and related dynamics of the deprotonated chromophore.

Direct excitation of the deprotonated chromophore of either the $Ca^{2+}$-free or bound REX-GECO1 leads to a prompt rise (within the instrument response function, measured as the cross-correlation time of ~140 fs between the fs actinic pump and probe pulses) and a subsequent triple exponential decay of $S_1$ species, which can be assigned to orientational relaxation (a few ps to ~20 ps), local environment with H-bonding network rearrangement (tens of ps),[5,6] and a combination of radiative and nonradiative downward transitions (hundreds of ps to ~1 ns).[1,7] The larger mismatch between ESA and SE intensity dynamics of the $Ca^{2+}$-bound REX-GECO1 (Figure S5d) than $Ca^{2+}$-free case (Figure S5c) following 570 nm pump could be due to the excitation of some mixed protonated/deprotonated species from the ground state ($S_0$), particularly for the $Ca^{2+}$-bound biosensor (Figure S1b).

Moreover, the ~17 ps decay component of the excited deprotonated chromophore in the $Ca^{2+}$-free biosensor after 570 nm excitation (Figure S5c) is significantly longer than the corresponding decay component of the excited protonated chromophore after 480 nm excitation (4.6 ps, which could involve some faster ESPT components, see Figure 2c).[1,8] Strikingly, the ESA band in the latter case exhibits a distinct rise component with a ~1.9 ps time constant. The existence of a rapidly accessible lower-lying electronic state than the initially populated Franck-Condon state is thus inferred,[8,9] corroborated by the torsional-angle-dependent quantum calculations and MD simulations (see Figure 3). Beyond ~10 ps, the similar decay components of the 532 nm ESA band and 592 nm SE band of the $Ca^{2+}$-free biosensor chromophore after 480 nm excitation mostly reflect the common vibrational cooling processes in $S_1$ (see Figure 2c, and above) and other nonradiative/radiative energy dissipation pathways that can decrease the excited-state chromophore population, which includes the inhomogeneous subpopulations that contribute to various TA spectral features.

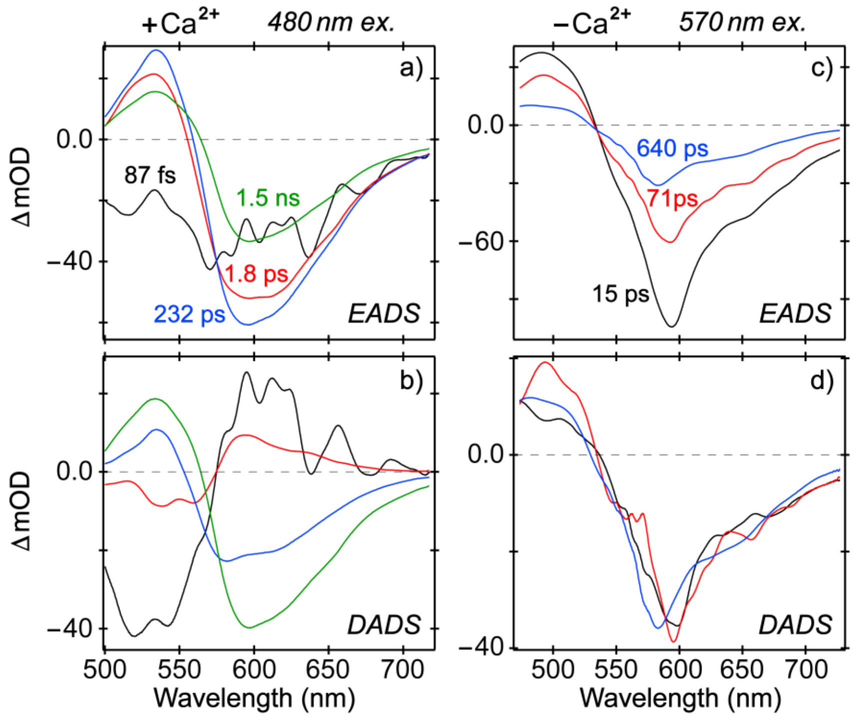


**Figure S6.** Global analysis of the time-resolved TA spectra of the $Ca^{2+}$-bound REX-GECO1 biosensor after 480 nm excitation in pH=7 buffer solution yields the (a) evolution-associated difference spectra (EADS) and (b) decay-associated difference spectra (DADS). The kinetic scheme following a sequential model leads to black→red→blue→green traces with their respective lifetimes denoted. The same analysis of the $Ca^{2+}$-free REX-GECO1 biosensor after 570 nm excitation in pH=7 buffer solution yields the (c) EADS and (d) DADS. The sequential model leads to black→red→blue traces with their associated lifetimes. The zero OD change is marked by the horizontal dashed gray line.

Notably with the 480/570 nm pump, only the pure protonated/deprotonated species of the $Ca^{2+}$-bound/free biosensor chromophores are predominantly excited (see Figure S1b). Therefore, global analysis with a sequential kinetic model was performed on these fs-TA data to uncover spectral features and mechanistic insights that are specific to the chromophore's protonation state, largely free from the overlapping bands of mixed species

(especially with other excitation wavelengths). The EADS and DADS of the $Ca^{2+}$-bound REX-GECO1 biosensor (Figure S6a and b) clearly show a spectroscopic transition from an SE band at ~530 nm to an SE band at ~600 nm, indicative of an ESPT process that converts the chromophore from its protonated form to deprotonated form. In contrast, this dynamics pattern was not retrieved from the $Ca^{2+}$-free biosensor spectra after 570 nm excitation since the starting chromophore species are already deprotonated (B form), hence a dominant excited-state species with concomitantly decaying ESA and SE bands (see Figure S6c with an isosbestic point, substantiating a dominant transient electronic state B*). The processes corresponding to the three characteristic time constants have been assigned on the basis of the probe-dependent TA data analysis following Figure S5 (see above).

In particular, the tens of ps (71 ps in Figure S6c) process exhibits a blueshift of the SE band (see the red→blue traces in Figure S6c and d), suggesting that local environment rearrangement modifies the chromophore's potential energy surface to result in a slightly increased energy gap between $S_1$ and $S_0$ states in this case. Notably, in contrast to chromophores in solution that typically displays a redder SE peak than the steady-state fluorescence peak (e.g., anionic HPTS in water, 522 nm > 510 nm; anionic 3-nitrotyrosine in water, 650 nm > 520 nm; anionic *p*-HBDI in water, 525 nm > 505 nm),[8-10] the chromophore embedded in a protein pocket exhibits a bluer SE peak than the fluorescence peak instead (e.g., inside GFP-S65T/S205V, 517 nm < 524 nm; inside sfGFP, 505 nm < 510 nm).[11,12] Such an opposite trend highlights the differences in chromophore-environment interactions that lead to characteristic stabilization effects of the $S_1$ and $S_0$ state energies during the photoinduced chromophore's structural and energy relaxation in the non-equilibrium regime.[1,13]

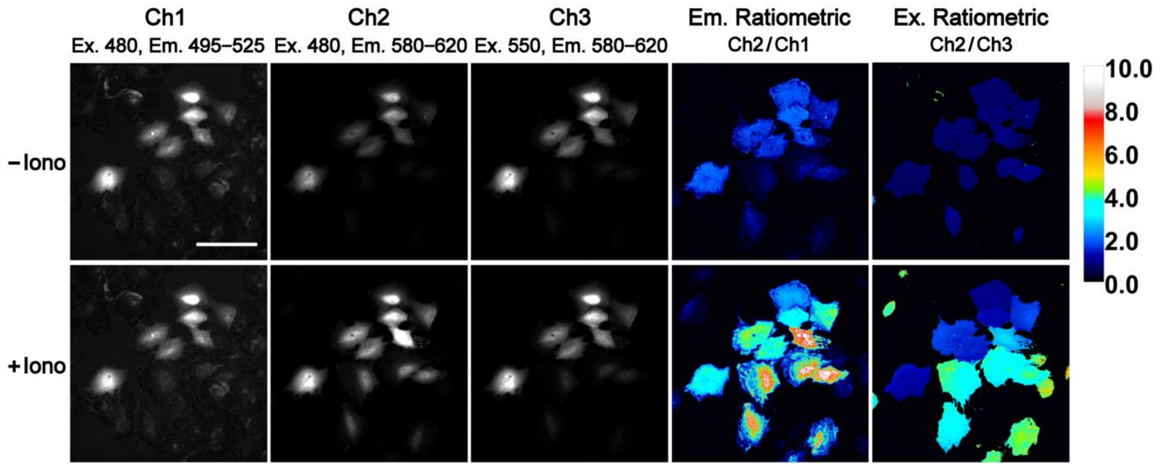


**Figure S7.** Contrasting the emission- and excitation-ratiometric imaging of the REX-GECO1 biosensor. In an expanded plot of Figure 4a (see main text), the third imaging channel data (Ch3 with excitation at 550 nm and emission at 580–620 nm) and the resultant excitation-ratiometric signal (Ch2/Ch3) are shown in the third and fifth columns, respectively. Scale bar = 100 μm. Direct comparison between the fourth and fifth columns shows that the emission-ratiometric method using REX-GECO1 leads to a higher dynamic range for cell imaging (see below).[14]

Notably, Figure 4a (main text) and Figure S7 (an expanded figure for Figure 4a) were both acquired in focus as the edges of the cells (pseudopodia) can be clearly observed. For the time-lapse imaging data using a pinhole size >10 AU (with a point spread function in Z axis >20 μm, large enough to cover the thickness of attached cells), the optical sectioning effect of confocal microscopy is no longer pronounced. We therefore employed the sharp observation of pseudopodia as the criterion for focusing the cells in our protocols (see Section 3.2 in main text for details) during the time-lapse imaging experiments for both confocal and wide-field microscopy setups.

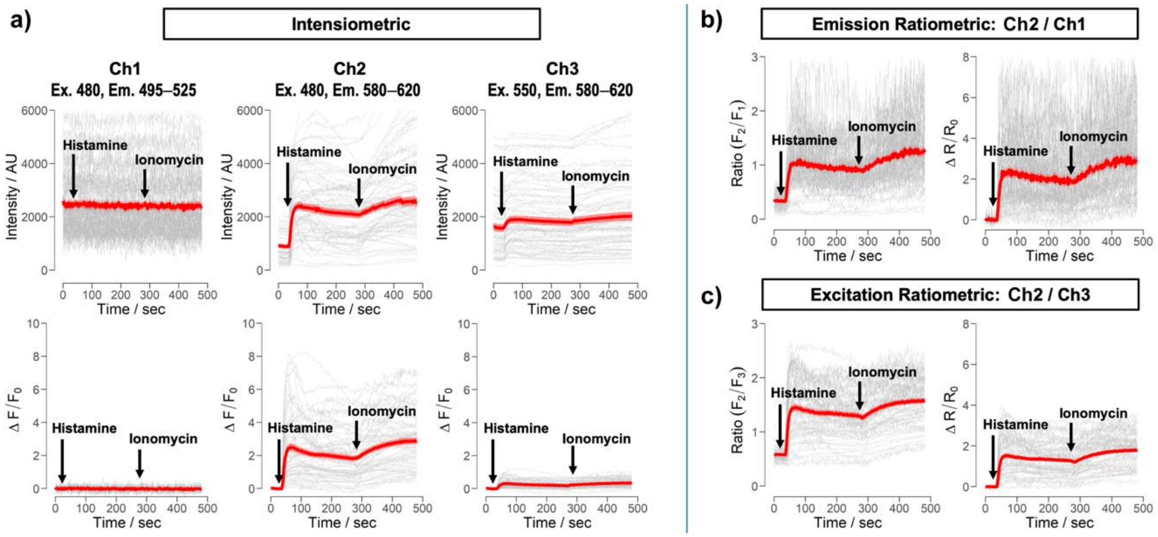


**Figure S8.** Raw experimental data and change in fluorescence signal at each individual imaging channel. (a) The intensiometric signal intensities (upper panels) and the associated fluorescence intensity changes ($\Delta F/F_0$) are displayed for Ch1, Ch2, and Ch3, respectively. Traces for individual cells (gray) lead to mean value for all the ROIs (red) and the standard error of the mean (s.e.m., pink shade around the red curve). On the right side, fluorescence signal intensity ratios (left) and the red/green emission ratio changes (right) for the (b) emission-ratiometric method and (c) excitation-ratiometric method demonstrate the ratiometric sensing capability of REX-GECO1 in HeLa cells.

Note that all the instrumentation parameters were kept the same as those for data collection in Figure 4 (main text) and Figure S7 above, except for a lower laser intensity to avoid photobleaching, a lower line average repetition number to increase the frame rate, and an altered gain voltage. A total of 70 cells were recorded and pooled from 3 replications to generate the traces. The green channel signal (Ch1) remains detectable with a sufficient signal-to-noise ratio (SNR, see Figure S10 below). During data acquisition, the laser power, gain values, scanning speed, and pinhole size were optimized to minimize photobleaching

effect as well as to maximize the photon collection at the frame rate of 0.5 Hz. Under such settings, with an emitting power of 4% at 480 nm (see Section 3.2 in main text), the raw intensity value of Ch1 is comparable, if not higher, than that of Ch2 (Figure S8a, upper panels). During data analysis, cells with intensity in Ch1 below a common threshold value of 500 were excluded; we did not observe distinct "high" and "low" intensity populations. When the intensity values of Ch1 after background subtraction are plotted, the signal from Ch1 is well separated from the background (Figure S8a, upper-left panel).

Furthermore, the measurement and calculation of $\Delta R/R_0$ normalized the effect of raw intensity values on the ratio. We thus conclude that the variation of Ch2/Ch1 emission ratios (Figure S8b) is largely attributed to the intrinsic low brightness of Ch1, rather than improper instrumentation setting, as is suggested by the SNR analysis in the lower panels of Figure S10 (see below). There also exists the variations of response from cell to cell, which could be roughly estimated by gray traces of the less noisy channels (Ch2 and Ch3) in Figure S8. With Ch1 as the denominator of large variation, it is not surprising that such noise is translated to the resulting ratio $F_2/F_1$ and the derived $\Delta R/R_0$ (Figure 4b).

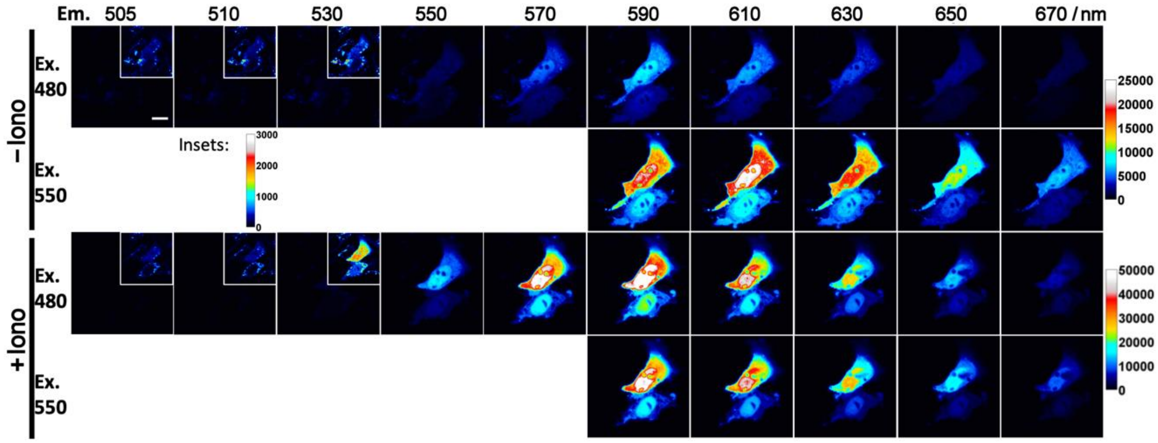


**Figure S9.** Manual λ-scan performed on HeLa cells expressing REX-GECO1 before (upper two rows) and after (lower two rows) the histamine and ionomycin (Iono) treatment with 480 or 550 nm excitation. Scale bar = 10 μm. In the green channels (Ex. 480 nm, Em. 505, 510, and 530 nm, see the insets of three upper left cells), the autofluorescence is comparable to the biosensor signal. The collection window was shifted across the spectrum with a 20 nm increment (except the first one at 505 nm).

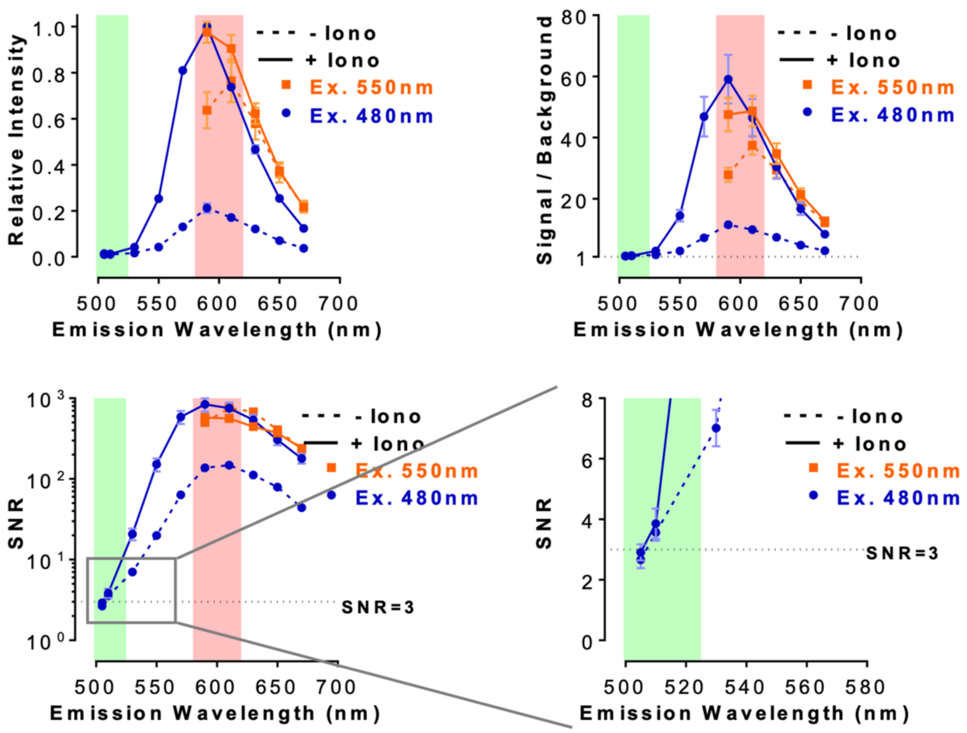


**Figure S10.** Manual λ-scan imaging data analysis (with mean filtering) on HeLa cells expressing REX-GECO1 before (dashed curves) and after (solid curves) ionomycin treatment with 480 nm (blue) or 550 nm (orange) excitation. A total of 26 ROIs from 6 cells were recorded, and the data are represented as mean ± s.e.m. The selected wavelength ranges of the green (495–525 nm) and red (580–620 nm) emission channels are highlighted by the light green and red shades, respectively. Note that in the enlarged plot (lower right panel), weak emission signal in the green channel satisfies the common SNR>3 criteria.[15] This figure also provides the comparison of brightness across the fluorescence spectrum.

## **SM References**


1. C. Fang and L. Tang, *Annu. Rev. Phys. Chem.*, 2020, **71**, 239-265.
2. M. J. Frisch, G. W. Trucks, H. B. Schlegel, G. E. Scuseria, M. A. Robb, J. R. Cheeseman, G. Scalmani, V. Barone, G. A. Petersson, H. Nakatsuji, X. Li, M. Caricato, A. V. Marenich, J. Bloino, B. G. Janesko, R. Gomperts, B. Mennucci, H. P. Hratchian, J. V. Ortiz, A. F. Izmaylov, J. L. Sonnenberg, D. Williams-Young, F. Ding, F. Lipparini, F. Egidi, J. Goings, B. Peng, A. Petrone, T. Henderson, D. Ranasinghe, V. G. Zakrzewski, J. Gao, N. Rega, G. Zheng, W. Liang, M. Hada, M. Ehara, K. Toyota, R. Fukuda, J. Hasegawa, M. Ishida, T. Nakajima, Y. Honda, O. Kitao, H. Nakai, T. Vreven, K. Throssell, J. J. A. Montgomery, J. E. Peralta, F. Ogliaro, M. J. Bearpark, J. J. Heyd, E. N. Brothers, K. N. Kudin, V. N. Staroverov, T. A. Keith, R. Kobayashi, J. Normand, K. Raghavachari, A. P. Rendell, J. C. Burant, S. S. Iyengar, J. Tomasi, M. Cossi, J. M. Millam, M. Klene, C. Adamo, R. Cammi, J. W. Ochterski, R. L. Martin, K. Morokuma, O. Farkas, J. B. Foresman and D. J. Fox, *Gaussian 16, Revision A.03*, Gaussian, Inc., Wallingford, CT, 2016.
3. J. T. M. Kennis, D. S. Larsen, I. H. M. van Stokkum, M. Vengris, J. J. van Thor and R. van Grondelle, *Proc. Natl. Acad. Sci. U. S. A.*, 2004, **101**, 17988-17993.
4. J. J. Snellenburg, S. Laptenok, R. Seger, K. M. Mullen and I. H. M. van Stokkum, *J. Stat. Softw.*, 2012, **49**, 1-22.
5. M. Maroncelli and G. R. Fleming, *J. Chem. Phys.*, 1987, **86**, 6221-6239.
6. W. Liu, Y. Wang, L. Tang, B. G. Oscar, L. Zhu and C. Fang, *Chem. Sci.*, 2016, **7**, 5484-5494.
7. S. R. Meech, *Chem. Soc. Rev.*, 2009, **38**, 2922-2934.

8. L. Tang, L. Zhu, Y. Wang and C. Fang, *J. Phys. Chem. Lett.*, 2018, **9**, 4969-4975.

9. M. A. Taylor, L. Zhu, N. D. Rozanov, K. T. Stout, C. Chen and C. Fang, *Phys. Chem. Chem. Phys.*, 2019, **21**, 9728-9739.

10. L. Tang and C. Fang, *J. Phys. Chem. B*, 2019, **123**, 4915-4928.

11. L. Tang, Y. Wang, L. Zhu, K. Kallio, S. J. Remington and C. Fang, *Phys. Chem. Chem. Phys.*, 2018, **20**, 12517–12526.

12. B. G. Oscar, L. Zhu, H. Wolfendeen, N. D. Rozanov, A. Chang, K. T. Stout, J. W. Sandwisch, J. J. Porter, R. A. Mehl and C. Fang, *Front. Mol. Biosci.*, 2020, **7**, 131.

13. C. Fang, L. Tang and C. Chen, *J. Chem. Phys.*, 2019, **151**, 200901.

14. J. Wu, A. S. Abdelfattah, L. S. Miraucourt, E. Kutsarova, A. Ruangkittisakul, H. Zhou, K. Ballanyi, G. Wicks, M. Drobizhev, A. Rebane, E. S. Ruthazer and R. E. Campbell, *Nat. Commun.*, 2014, **5**, 5262.

15. Y. Shen, H. Dana, A. S. Abdelfattah, R. Patel, J. Shea, R. S. Molina, B. Rawal, V. Rancic, Y.-F. Chang, L. Wu, Y. Chen, Y. Qian, M. D. Wiens, N. Hambleton, K. Ballanyi, T. E. Hughes, M. Drobizhev, D. S. Kim, M. Koyama, E. R. Schreiter and R. E. Campbell, *BMC Biol.*, 2018, **16**, 9.
