# Supplementary material for: Switching between Ultrafast Pathways Enables a Green-Red Emission Ratiometric Fluorescent-Protein-Based Ca2+ Biosensor
Source: Int J Mol Sci. 2021 Jan 5;22(1):445. doi: 10.3390/ijms22010445 (PMC7794744; doi:10.3390/ijms22010445)
Supplement: Supplementary file 1 [file ijms-22-00445-s001.zip › Video_S1_REXGECO_ImagingSZ.pptx]

## Slide 1
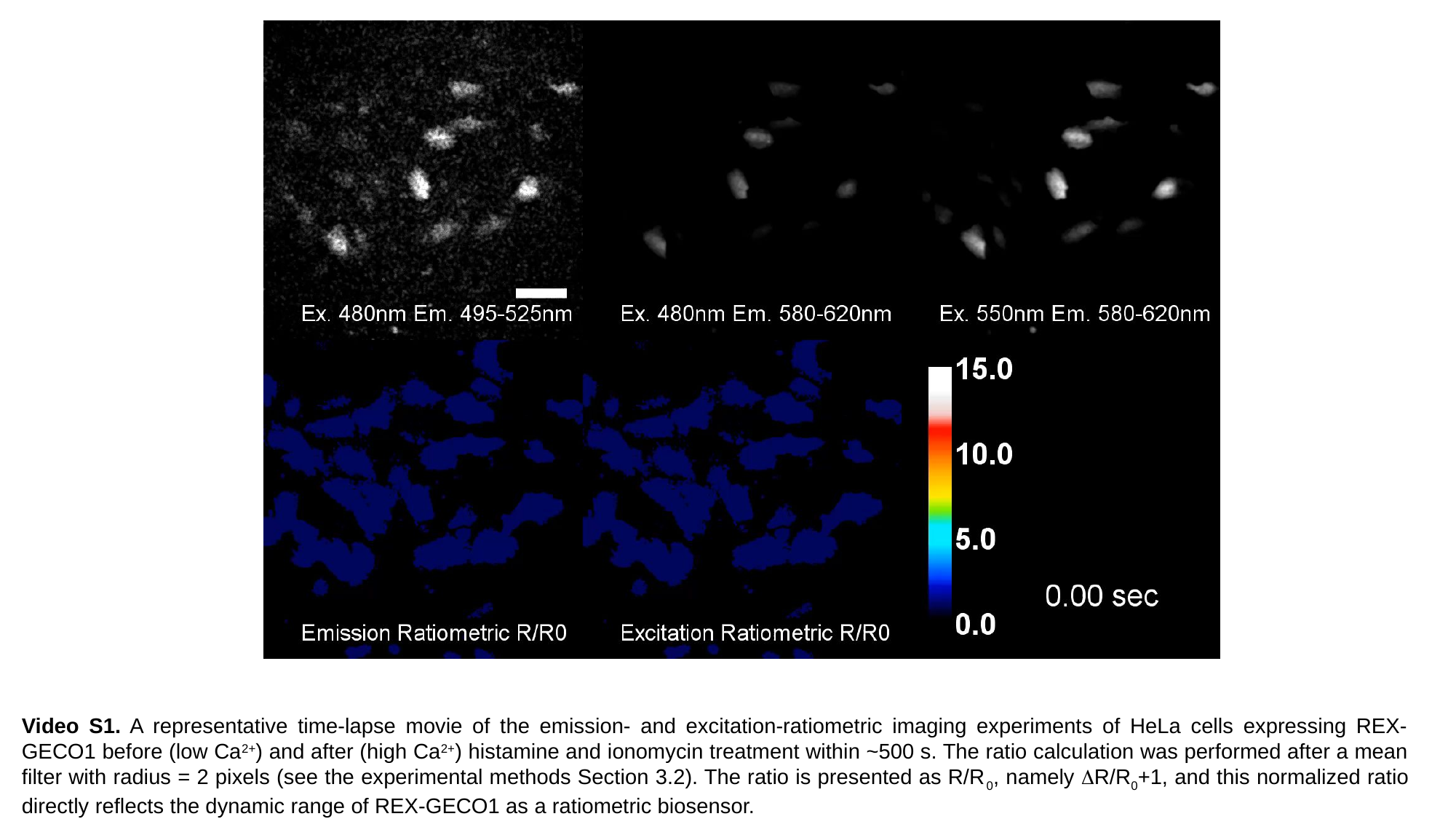

Video S1. A representative time-lapse movie of the emission- and excitation-ratiometric imaging experiments of HeLa cells expressing REX-GECO1 before (low Ca2+) and after (high Ca2+) histamine and ionomycin treatment within ~500 s. The ratio calculation was performed after a mean filter with radius = 2 pixels (see the experimental methods Section 3.2). The ratio is presented as R/R0, namely DR/R0+1, and this normalized ratio directly reflects the dynamic range of REX-GECO1 as a ratiometric biosensor.
